# Young adults are more vulnerable to chronic sleep deficiency and recurrent circadian disruption than older adults

**DOI:** 10.1038/s41598-018-29358-x

**Published:** 2018-07-23

**Authors:** Kirsi-Marja Zitting, Mirjam Y. Münch, Sean W. Cain, Wei Wang, Arick Wong, Joseph M. Ronda, Daniel Aeschbach, Charles A. Czeisler, Jeanne F. Duffy

**Affiliations:** 1 Division of Sleep and Circadian Disorders, Departments of Medicine and Neurology, Brigham and Women’s Hospital, Boston, MA 02115 USA; 2 Division of Sleep Medicine, Harvard Medical School, Boston, MA 02115 USA; 3 Present Address: Charité University Medicine Berlin, Institute of Physiology, Group Sleep Research and Clinical Chronobiology, Berlin, Germany; 4 Present Address: School of Psychological Sciences and Monash Institute of Cognitive and Clinical Neurosciences, Monash University, Clayton, Australia; 5 Present Address: Division of Sleep and Human Factors Research, Institute of Aerospace Medicine, German Aerospace Center, Cologne, Germany

## Abstract

More than a third of US adults report fewer than 6 hours of sleep a night, making chronic sleep restriction a growing public health concern. Sleep curtailment is associated with an increase in industrial accidents, motor vehicle accidents, medical and other occupational errors. Young adults are more vulnerable to acute sleep deprivation than older adults, but less is known about how young vs. older adults respond to the more commonly experienced chronic sleep restriction. To test the hypothesis that young adults are more vulnerable to chronic sleep loss than older adults, we compared data from young and older adults who underwent three weeks of chronic sleep restriction (equivalent to 5.6 hours/24 hours) combined with recurrent circadian disruption in an experiment that enabled us to separate the influences of the sleep-wake homeostatic process, the circadian timing system, and the chronic sleep deficit. We found that while young and older adults reported similar levels of subjective sleepiness, objective measures of sleepiness revealed that young adults were more vulnerable and had more attentional failures than the older adults. These results have important public health implications, particularly related to prevention of sleep-related motor vehicle crashes in young drivers. Further research is needed to understand the neurobiological basis of these age-related differences.

## Introduction

Studies in both humans and animals have unequivocally demonstrated profound differences in sleep architecture across the life span. The depth of sleep, as measured by electroencephalographic (EEG) slow wave activity (SWA, 0.5–4.5 Hz) during slow wave sleep (SWS), is greatest in childhood, remains high during adolescence and young adulthood, and decreases with age^1–4^.

Because SWS and SWA are considered to reflect a sleep homeostatic process^5^, it raises the question as to whether the age-related decrease in sleep duration, SWS, and SWA are signs of reduced homeostatic sleep need^6–8^. An alternative interpretation is that sleep need remains unchanged across the lifespan but the ability of the aging brain to respond to sleep need or to dissipate/release sleep pressure through the production of slow waves, is compromised^9^. In other words, do young people need more sleep or is their need for sleep unchanged, and/or are young adults able to fulfill their sleep need whereas older adults are no longer able to do so?.

Both of these interpretations have evidence to support them^8,10–12^. However, methodological differences and differences in study design (acute vs. chronic sleep deprivation), and study participants (e.g., health status, circadian timing) have made it challenging to compare across studies and answer this question definitively. It may also be that a combination of reduced sleep pressure and compromised ability to release it underlie the reduced SWS in older as compared to young adults.

One way to determine whether there is a reduction in sleep need or instead a reduction in the ability to sleep with age is to focus on waking performance and the ability to sustain wakefulness, given that it is well-established that chronic insufficient sleep duration and/or reduced sleep quality will inevitably lead to poorer daytime performance^13–15^. Patients with sleep disorders such as sleep apnea have disturbed nocturnal sleep leading to excessive daytime sleepiness and changes in cognitive performance^16,17^, similar to the result of inadequate/insufficient nocturnal sleep that results in daytime sleepiness and performance decrements. If age-related changes in sleep, observed even among extremely healthy older adults^18,19^, are due to an inability to dissipate homeostatic sleep need, then that impairment ought to manifest as poorer performance when healthy older adults are sleep restricted or subject to acute sleep loss paradigms.

In previous studies, we and others have found that healthy young adults are more vulnerable to acute sleep deprivation than older adults, as measured by objective assessments of both sleepiness and performance^7,20–22^. While acute total sleep deprivation is often used to probe the impact of sleep loss in the laboratory, people much more commonly experience both chronic sleep deficiency and irregular timing of the sleep-wake cycle in their everyday lives. Therefore, the purpose of the current study was to test the hypothesis that healthy young adults are more vulnerable than older adults to chronic sleep restriction combined with recurrent circadian disruption using three measures: subjective and objective sleepiness and waking cognitive performance.

## Results

### Baseline

**Figure 1:**
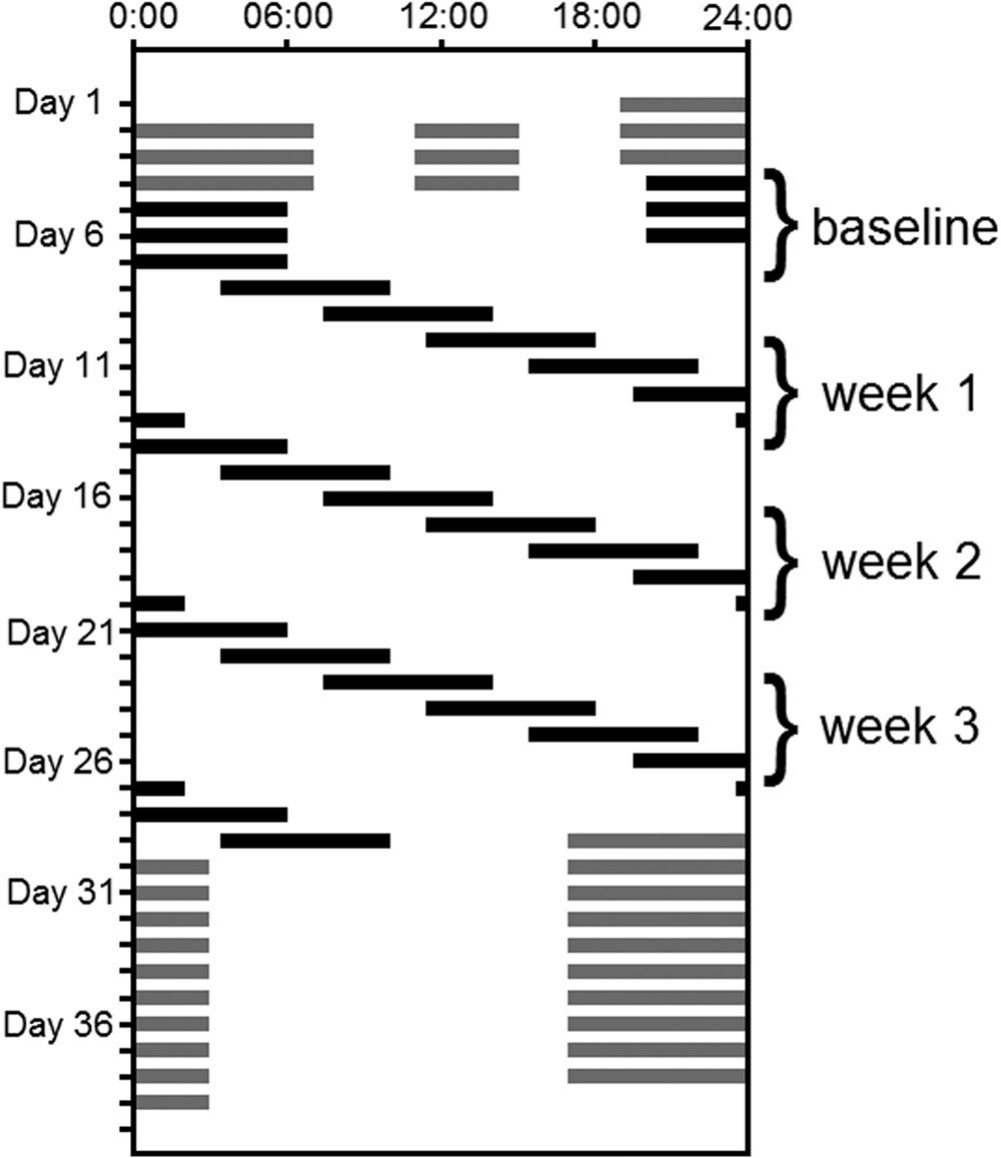
Raster plot of study schedule. Time of day is presented across the horizontal axis, and subsequent days of the experiment are plotted beneath each other along the vertical axis from top to bottom. Solid bars indicate scheduled sleep episodes, open bars indicate wake episodes. The first segment of the inpatient study was a sleep extension segment during which participants spent 16 hours in bed/day (12 hours each night and 4 hours each day, indicated by grey bars) for three days, followed by 3 baseline 24-hour days with 10 hours in bed/night. This was followed by three weeks of chronic sleep restriction-forced desynchrony (CSR-FD) during which participants were scheduled on a 28-hour “day” with 6.5 hour sleep opportunities (equivalent to 5.6 hours per 24 hours). Following the CSR-FD portion of the protocol, participants underwent a recovery segment for 10 24-hour days with 10 hours in bed/night.

At baseline (Fig. 1), no significant differences between the two age groups were found for any of the four variables: Karolinska Sleepiness Scale (KSS: *F*_*1*,*1155*_ = *0*.*53*, *p* = *0*.*4653*); the number of Psychomotor Vigilance Task (PVT) lapses (*F*_*1*,*110*_ = *0*.*87*, *p* = *0*.*3542*); the number of Slow Eye Movements (SEMs: *F*_*1*,*1010*_ = *0*.*74*, *p* = *0*.*3894*); or the number of inadvertent sleep epochs (SLEEPs: *F*_*1*,*1010*_ = *0*.*94*, *p* = *0*.*3324*). Thus, baseline was not included as a covariate in the final statistical models.

### Subjective Sleepiness

**Figure 2:**
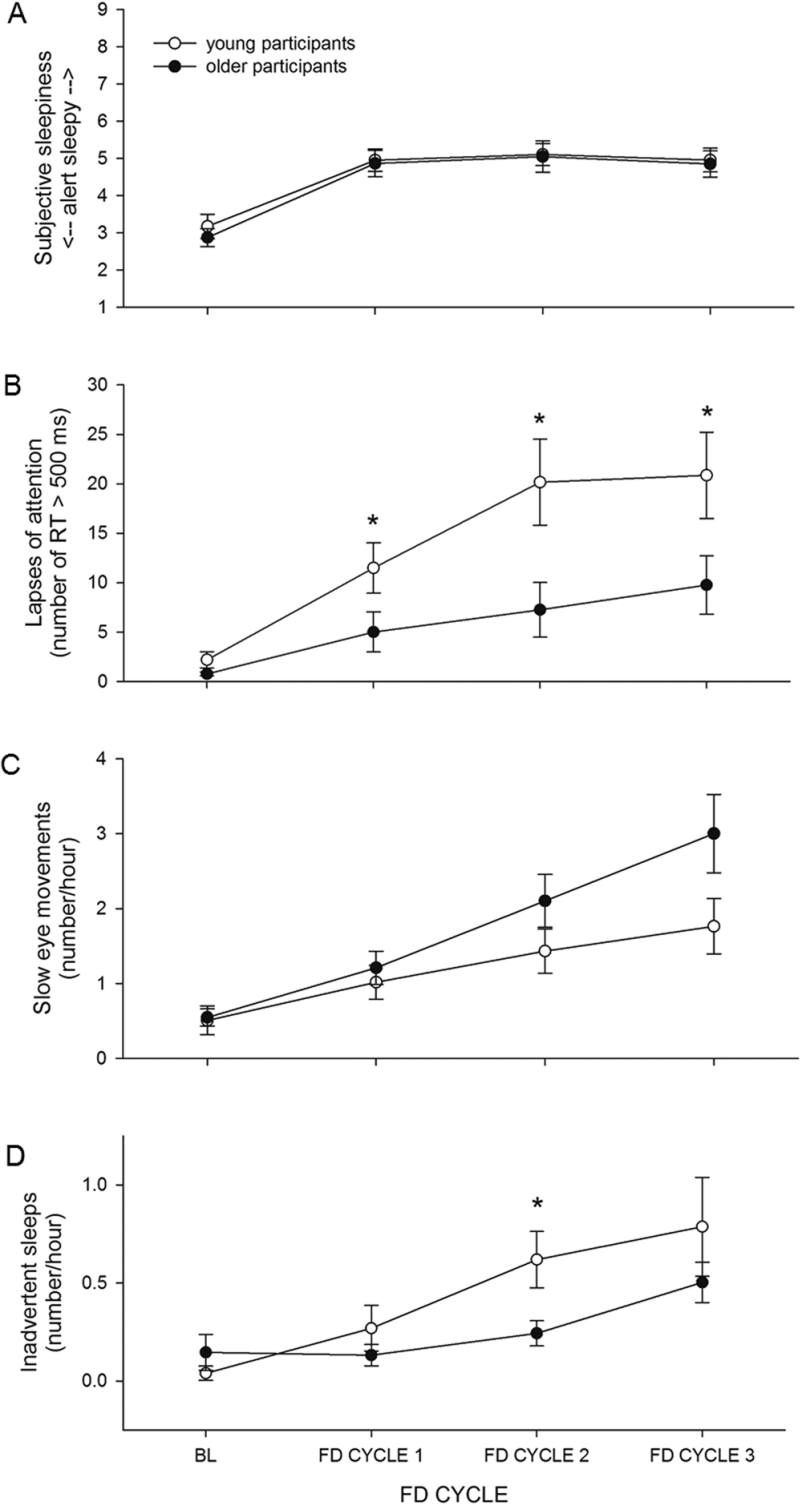
FD CYCLE x AGE. (**A**) Subjective sleepiness (n.s.), (**B**) lapses of attention (p < 0.0001), (**C**) SEMs (p = 0.0054), and (**D**) inadvertent sleep (p < 0.0001) data averaged for the 3 baseline days and for each of the three FD cycles. Mean ± s.e.m. (n = 12 per group except for PVT lapses where n = 11 for older participants) are shown. *Indicates a significant p-value in post-hoc tests.

Mixed-model analysis revealed a significant main effect of Forced Desynchrony (FD) CYCLE on subjective sleepiness as measured by KSS score (*F*_*2*,*16*,*300*_ = *30*.*60*, *p* < *0*.*0001*), with participants reporting feeling sleepiest during the second FD cycle. There was no significant main effect of AGE and the interaction of FD CYCLE and AGE was also not significant (Fig. 2A).

**Figure 3:**
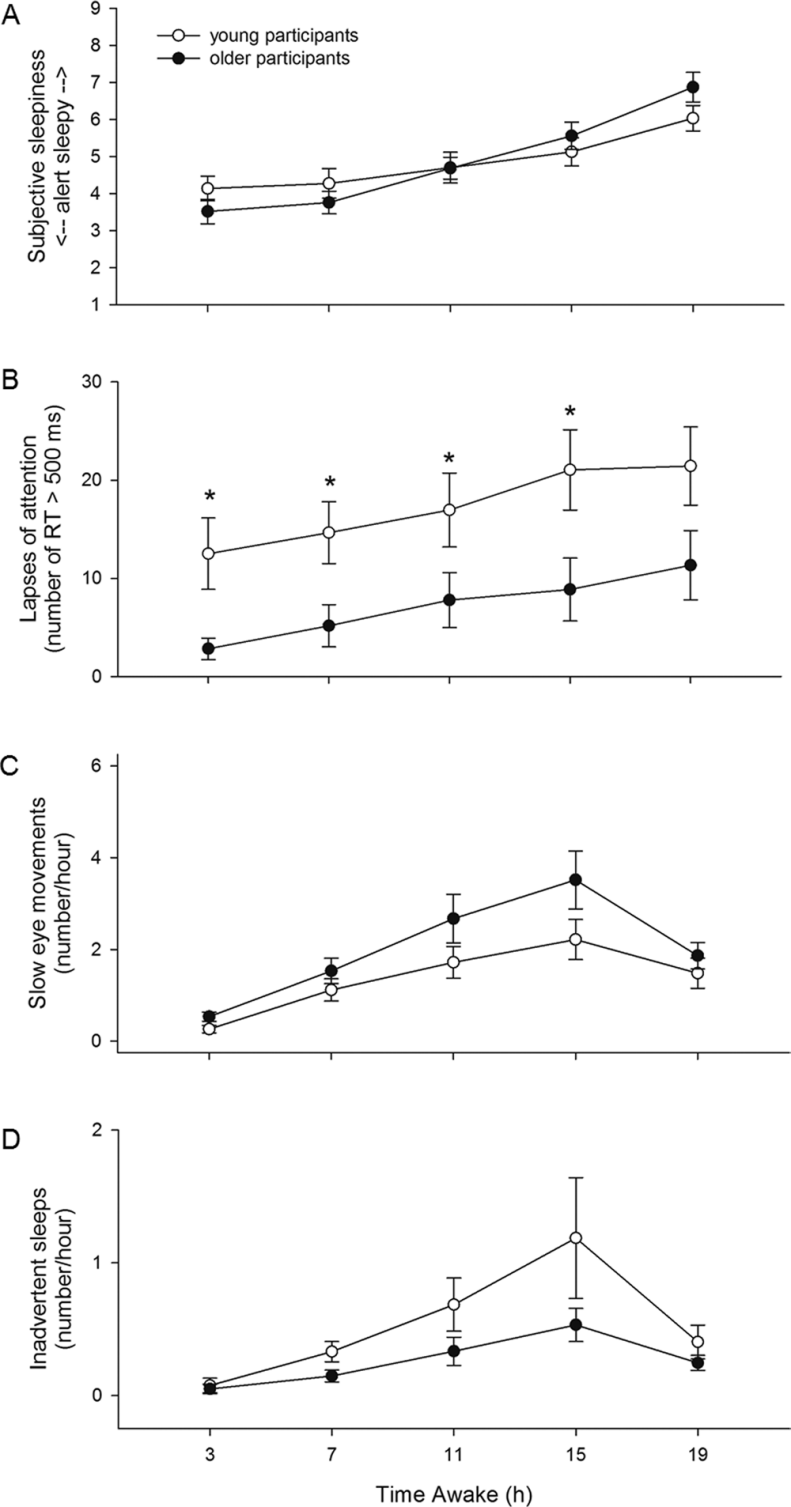
TIME AWAKE x AGE. (**A**) Subjective sleepiness (p < 0.0001), (**B**) lapses of attention (p < 0.0001), (**C**) SEMs (p = 0.0322), and (**D**) inadvertent sleeps (p < 0.0001) data averaged in 4-hour awake bins across the three FD cycles. Mean ± s.e.m. (n = 12 per group except for PVT lapses where n = 11 for older participants) are shown. *Indicates a significant p-value in post-hoc tests.

There was a significant main effect of TIME AWAKE on subjective sleepiness (*F*_*4*,*16*,*300*_ = *1821*.*28*, *p* < *0*.*0001*), with participants reporting increased sleepiness the longer they were awake. There was an interaction of TIME AWAKE and AGE (*F*_*4*,*16*,*300*_ = *163*.*17*, *p* < *0*.*0001*), but post hoc analysis did not reveal significant differences in subjective sleepiness between the two age groups for any time awake bin (Fig. 3A).

**Figure 4:**
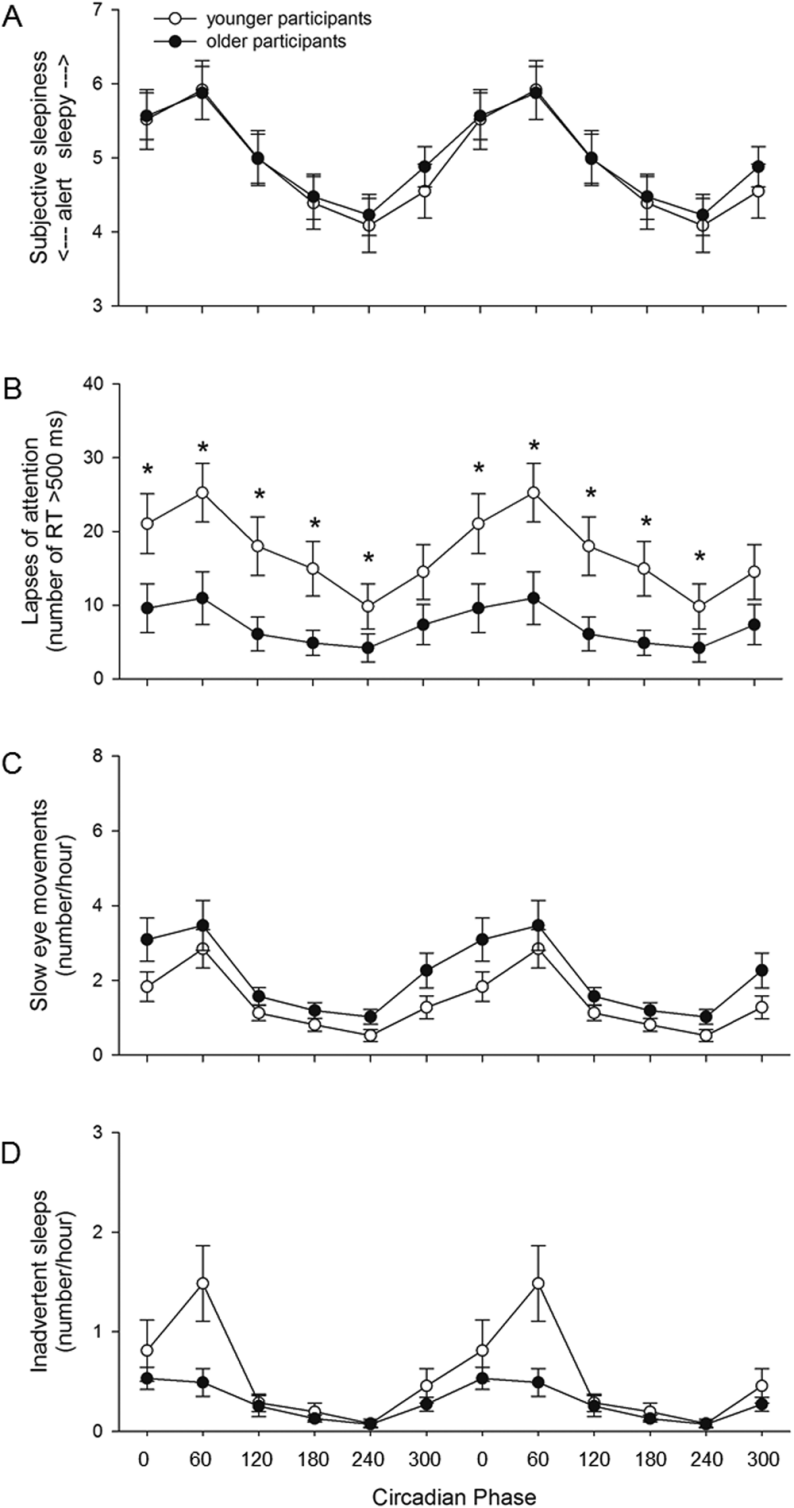
CIRCADIAN PHASE x AGE. (**A**) Subjective sleepiness (p < 0.0001), (**B**) lapses of attention (p < 0.0001), (**C**) SEMs (p = 0.0444), and (**D**) inadvertent sleeps (n.s.) data averaged in ~4 hour circadian phase bins (double plotted) across the three FD cycles. Mean ± s.e.m. (n = 12 per group except for PVT lapses where n = 11 for older participants) are shown. *Indicates a significant p-value in post-hoc tests.

CIRCADIAN PHASE also had a significant main effect on subjective sleepiness (*F*_*5*,*16*,*300*_ = *528*.*12*, *p* < *0*.*0001*), with the greatest levels of sleepiness reported at circadian phase 60° (spanning 2–6 hours after the time of the core body temperature minimum, corresponding to early biological morning). The interaction of CIRCADIAN PHASE and AGE was also significant (*F*_*5*,*16*,*300*_ = *8*.*45*, *p* < *0*.*0001*), but post hoc analysis did not reveal significant differences in subjective sleepiness between the two age groups for any circadian phase bin (Fig. 4A).

**Figure 5:**
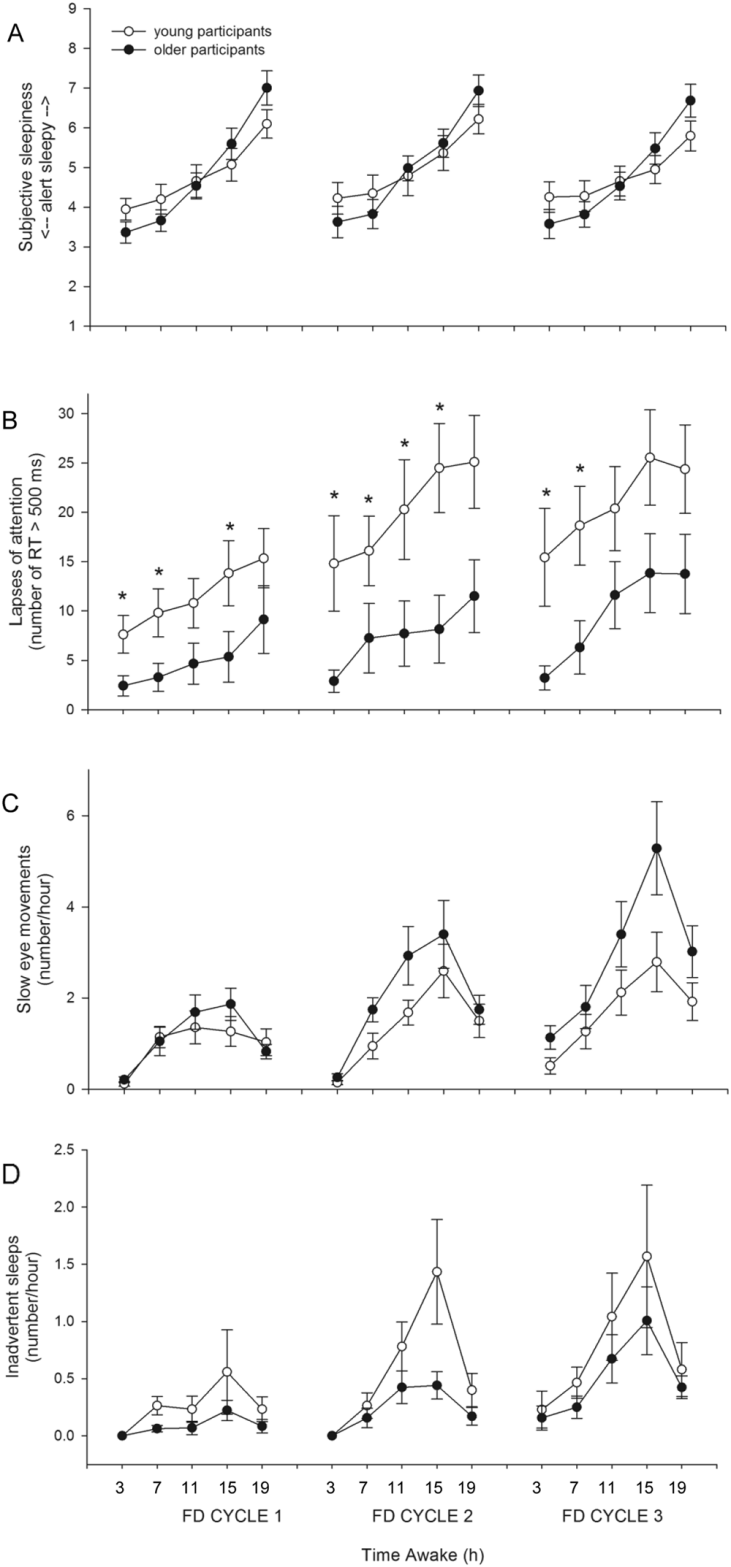
AGE x FD CYCLE x TIME AWAKE. (**A**) Subjective sleepiness (p = 0.0112), (**B**) lapses of attention (p < 0.0001), (**C**) SEMs (p = 0.0022), and (**D**) inadvertent sleeps (p < 0.0001) data averaged in 4-hour awake bins for each of the three FD cycles. Mean ± s.e.m. (n = 12 per group except for PVT lapses where n = 11 for older participants) are shown. *Indicates a significant p-value in post-hoc tests.

**Figure 6:**
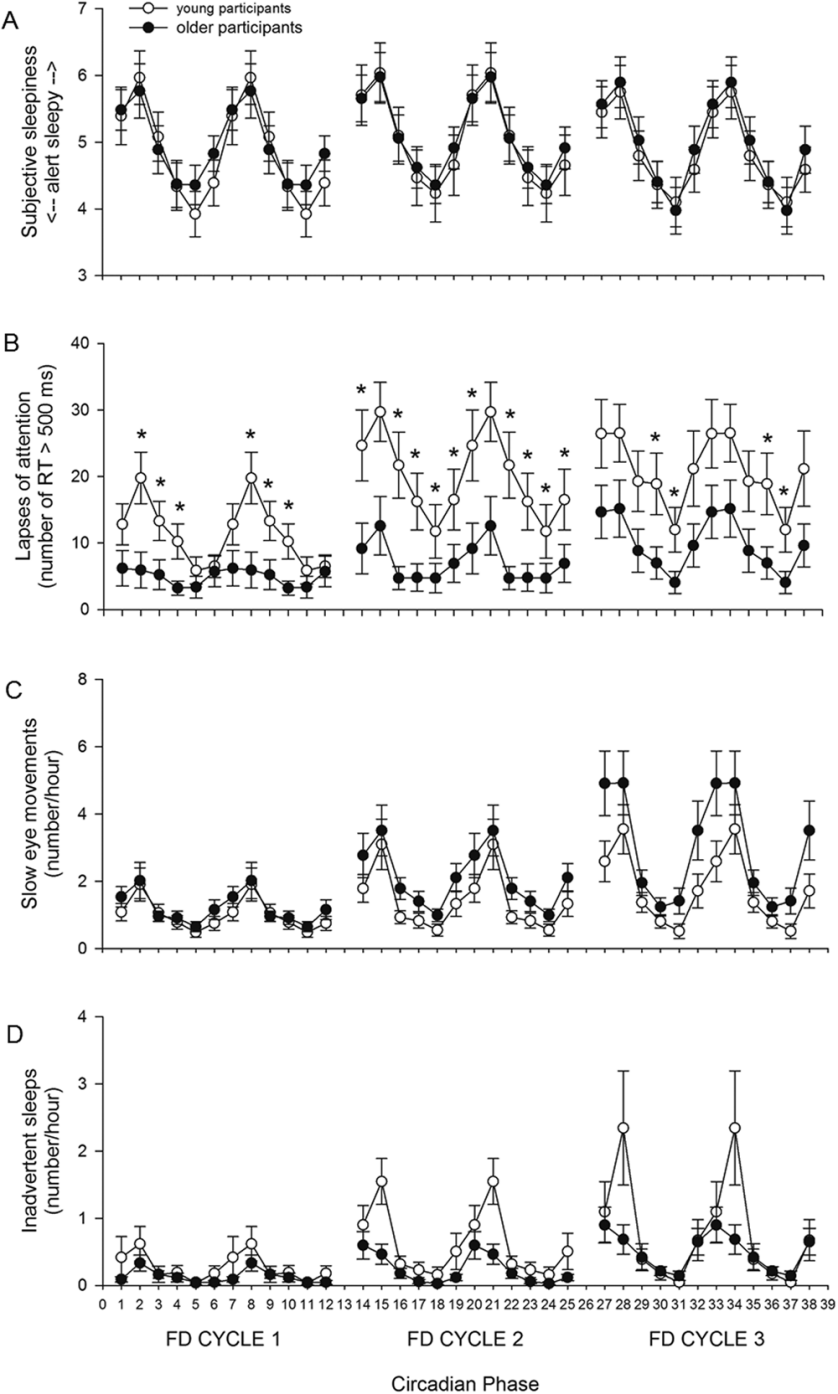
AGE x FD CYCLE x CIRCADIAN PHASE. (**A**) Subjective sleepiness (n.s.), (**B**) lapses of attention (p < 0.0001), (**C**) SEMs (n.s.), and (**D**) inadvertent sleeps (n.s.) data averaged in ~4-hour circadian phase for each of the three FD cycles. Mean ± s.e.m. (n = 12 per group except for PVT lapses where n = 11 for older participants) are shown. *Indicates a significant p-value in post-hoc tests.

There was a significant interaction between FD CYCLE, TIME AWAKE and AGE on subjective sleepiness (*F*_*8*,*16*,*300*_ = *2*.*47*, *p* = *0*.*0112*), but post hoc analysis did not show significant differences between the two age groups at any level of time awake and FD cycle (Fig. 5A). We did not test the interaction between FD CYCLE, CIRCADIAN PHASE and AGE (Fig. 6A). There was also a significant interaction between TIME AWAKE, CIRCADIAN PHASE, and AGE (*F*_*20*,*16*,*300*_ = *3*.*83*, *p* < *0*.*0001*), but again post hoc analysis did not show significant differences in subjective sleepiness between the age groups at any level of time awake and circadian phase.

### Sustained Attention

Mixed-model analysis revealed a significant main effect of FD CYCLE on the number of PVT lapses (*F*_*2*,*1829*_ = *481*.*16*, *p* < *0*.*0001*), with participants having more lapses with each successive FD cycle. There was also a significant main effect of AGE (*F*_*1*,*1829*_ = *9*.*84*, *p* = *0*.*0017*), with older participants having fewer lapses (Fig. 2B). The interaction of FD CYCLE and AGE was also significant (*F*_*2*,*1829*_ = *31*.*73*, *p* < *0*.*0001*), with young participants having significantly more lapses than older participants during each of the three FD cycles (*FD cycle 1*: *F*_*1*,*1829*_ = *8*.*62*, *p* = *0*.*0102*; *FD cycle 2*: *F*_*1*,*1829*_*12*.*45*, *p* = *0*.*0012*; *FD cycle 1*: *F*_*1*,*1829*_ = *8*.*62*, *p* = *0*.*0102*; Fig. 2B).

TIME AWAKE had a significant main effect on the number of lapses (*F*_*4*,*1829*_ = *382*.*11*, *p* < *0*.*0001*), with participants having more lapses the longer they were awake. There was a significant interaction of TIME AWAKE and AGE (*F*_*4*,*1829*_ = *59*.*04*, *p* < *0*.*0001*), with young participants having significantly more lapses than older participants in the 3-hour (*F*_*1*,*1829*_ = *16*.*86*, *p* < *0*.*0001*), 7-hour (*F*_*1*,*1829*_ = *11*.*09*, *p* = *0*.*0045*), 11-hour (*F*_*1*,*1829*_ = *8*.*12*, *p* = *0*.*0220*), and 15-hour (*F*_*1*,*1829*_ = *8*.*82*, *p* = *0*.*0150*) time awake bins (Fig. 3B).

CIRCADIAN PHASE also had a significant main effect on the number of lapses (*F*_*5*,*1829*_ = *278*.*27*, *p* < *0*.*0001*, Fig. 4B), with the greatest number of lapses occurring at circadian phase 60° (spanning 2–6 hours after the core body temperature minimum, corresponding to early biological morning). There was a significant interaction of CIRCADIAN PHASE and AGE (*F*_*5*,*1829*_ = *21*.*76*, *p* < *0*.*0001*), with both age groups showing a maximum number of lapses at circadian phase 60° and fewest lapses at circadian phase 240°, and young participants having significantly more lapses than older participants at circadian phase bins 0° (*F*_*1*,*1829*_ = *8*.*47*, *p* = *0*.*0220*), 60° (*F*_*1*,*1829*_ = *9*.*42*, *p* = *0*.*0132*), 120° (*F*_*1*,*1829*_ = *11*.*97*, *p* = *0*.*0030*), 180° (*F*_*1*,*1829*_ = *14*.*21*, *p* = *0*.*0012*), and 240° (*F*_*1*,*1829*_ = *9*.*24*, *p* = *0*.*0144*; Fig. 4B).

There was a significant interaction between FD CYCLE, TIME AWAKE, and AGE on the number of lapses (*F*_*8*,*1829*_ = *5*.*33*, *p* < *0*.*0001*). While in both age groups the number of lapses increased as time awake increased, the increase in lapses with time awake was gradual and consistent across FD cycle for older participants, but for young participants the magnitude of the increase in lapses with time awake was greater and reached its maximum during the second FD cycle. Young participants had significantly more lapses than older participants in the 3-hour (*F*_*1*,*1829*_ = *11*.*22*, *p* = *0*.*0120*), 7-hour (*F*_*1*,*1829*_ = *11*.*16*, *p* = *0*.*0135*), and 15-hour (*F*_*1*,*1829*_ = *8*.*88*, *p* = *0*.*0435*) time awake bins during the first FD cycle, in the 3-hour (*F*_*1*,*1829*_ = *22*.*66*, *p* < *0*.*0001*), 7-hour (*F*_*1*,*1829*_ = *10*.*82*, *p* = *0*.*0150*), 11-hour (*F*_*1*,*1829*_ = *10*.*50*, *p* = *0*.*0180*) and 15-hour (*F*_*1*,*1829*_ = *11*.*76*, *p* = *0*.*0090*) time awake bins during the second FD cycle, and in the 3-hour (*F*_*1*,*1829*_ = *16*.*65*, *p* < *0*.*0001*) and 7-hour (*F*_*1*,*1829*_ = *10*.*82*, *p* = *0*.*0150*) time awake bins of the third FD cycle (Fig. 5B).

There was also a significant interaction between FD CYCLE, CIRCADIAN PHASE, and AGE (*F*_*10*,*1829*_ = *14*.*79*, *p* < *0*.*0001*, Fig. 6B), with young participants having significantly more lapses than older participants at circadian phase bins 60° (*F*_*1*,*1829*_ = *13*.*32*, *p* = *0*.*0054*), 120° (*F*_*1*,*1829*_ = *10*.*37*, *p* = *0*.*0234*) and 180° (*F*_*1*,*1829*_ = *14*.*36*, *p* = *0*.*0036*) during the first FD cycle, at circadian phase bins 0° (*F*_*1*,*1829*_ = *11*.*29*, *p* = *0*.*0144*), 120° (*F*_*1*,*1829*_ = *18*.*92*, *p* < *0*.*0001*), 180° (*F*_*1*,*1829*_ = *14*.*82*, *p* = *0*.*0018*), 240° (*F*_*1*,*1829*_ = *11*.*22*, *p* = *0*.*0144*), 300° (*F*_*1*,*1829*_ = *9*.*92*, *p* = *0*.*0306*) during the second FD cycle, and at circadian phase bins 180° (*F*_*1*,*1829*_ = *12*.*82*, *p* = *0*.*0072*) and 240° (*F*_*1*,*1829*_ = *10*.*30*, *p* = *0*.*0252*) during the third FD cycle (Fig. 6B).

The interaction between TIME AWAKE, CIRCADIAN PHASE, and AGE was also significant (*F*_*20*,*1829*_ = *6*.*03*, *p* < *0*.*0001*), with young participants having significantly more lapses than older participants at circadian phase bins 0° (*F*_*1*,*1829*_ = *16*.*16*, *p* = *0*.*0018*), 60° (*F*_*1*,*1829*_ = *10*.*30*, *p* = *0*.*0390*), 120° (*F*_*1*,*1829*_ = *16*.*73*, *p* = *0*.*0013*), 180° (*F*_*1*,*1829*_ = *19*.*54*, *p* = *0*.*0003*), 240° (*F*_*1*,*1829*_ = *20*.*52*, *p* = *0*.*0002*, and 300° (*F*_*1*,*1829*_ = *13*.*47*, *p* = *0*.*0090*) at time awake bin 3-hour, at circadian phase bins 60° (*F*_*1*,*1829*_ = *15*.*05*, *p* = *0*.*0032*), 180° (*F*_*1*,*1829*_ = *18*.*58*, *p* = *0*.*0005*), and 240° (*F*_*1*,*1829*_ = *10*.*18*, *p* = *0*.*0420*) at time awake bin 7-hour, at circadian phase bins 120° (*F*_*1*,*1829*_ = *12*.*25*, *p* = *0*.*0150*), and 180° (*F*_*1*,*1829*_ = *13*.*47*, *p* = *0*.*0060*) at time awake bin 11-hour, and at circadian phase bin 120° (*F*_*1*,*1829*_ = *16*.*48*, *p* = *0*.*0016*) at time awake bin 15-hour.

### Slow Eye Movements

Mixed-model analysis revealed a significant main effect of FD CYCLE on the number of SEMs (*F*_*2*,*11*,*310*_ = *92*.*23*, *p* < *0*.*0001*), with participants having more epochs containing SEMs with each successive FD cycle. There was no main effect of AGE. There was a significant interaction of FD CYCLE and AGE (*F*_*2*,*11*,*310*_ = *5*.*23*, *p* = *0*.*0054*), but post hoc analysis did not reveal significant differences in the number of SEMs between the two age groups for any FD cycle (Fig. 2C).

There was a significant main effect of TIME AWAKE on the number of SEMs (*F*_*4*,*11*,*310*_ = *34*.*18*, *p* < *0*.*0001*). Participants had more SEMs the longer they were awake, although the greatest number was observed at the 15-hour time awake bin, and not at the 19-hour time awake bin. This may have been due to more frequent study staff-participant interactions during the hours immediately prior to bed time when participants were being readied for their sleep episode. There was also a significant interaction of TIME AWAKE and AGE (*F*_*4*,*11*,*310*_ = *2*.*64*, *p* = *0*.*0322*), but post hoc analysis did not reveal significant differences in the number of SEMs between the two age groups for any time awake bin (Fig. 3C).

CIRCADIAN PHASE had a significant main effect on the number of SEMs (*F*_*5*,*11*,*310*_ = *71*.*28*, *p* < *0*.*0001*), with the greatest number of SEMs occurring at circadian phase 60° (2–6 hours after the core body temperature minimum, corresponding to early biological morning). There was also a significant interaction of CIRCADIAN PHASE and AGE (*F*_*5*,*11*,*310*_ = *2*.*28*, *p* = *0*.*0444*), but post hoc analysis did not reveal significant differences in the number of SEMs between the two age groups for any circadian phase bin (Fig. 4C).

There was significant interaction between FD CYCLE, TIME AWAKE, and AGE on the number of SEMs (*F*_*8*,*11*,*310*_ = *3*.*02*, *p* = *0*.*0022*), but post hoc analysis did not show significant differences in the number of SEMs between the two age groups for any level of time awake and FD cycle (Fig. 5C). The interaction between FD CYCLE, CIRCADIAN PHASE, and AGE was not significant (Fig. 6C).

### Inadvertent Sleep

Mixed-model analysis revealed a significant main effect of FD CYCLE on the number of epochs containing inadvertent sleep (*F*_*2*,*11*,*310*_ = *1093*.*94*, *p* < *0*.*0001*), with participants having more inadvertent sleep with each successive FD cycle. The main effect of AGE was not significant, but there was a significant interaction of FD CYCLE and AGE (*F*_*2*,*11*,*310*_ = *19*.*61*, *p* < *0*.*0001*), with young participants having significantly more inadvertent sleeps than older participants during the second FD cycle (*F*_*1*,*11*,*310*_ = *12*.*74*, *p* = *0*.*0012*; Fig. 2D).

There was a significant main effect of TIME AWAKE on the number of inadvertent sleeps (*F*_*4*,*11*,*310*_ = *6*.*19*, *p* < *0*.*0001*). Similar to the SEM data, participants had more inadvertent sleep epochs the longer they were awake, although the greatest number were observed in the 15-hour time awake bin. There was also a significant interaction of TIME AWAKE and AGE (*F*_*2*,*11*,*310*_ = *26*.*15*, *p* < *0*.*0001*), but post hoc analysis did not reveal significant differences in the number of inadvertent sleeps between the two age groups for any time awake bin (Fig. 3D).

CIRCADIAN PHASE had a significant main effect on the number of inadvertent sleeps (*F*_*5*,*11*,*310*_ = *6*.*57*, *p* < *0*.*0001*), with the greatest number of sleeps occurring at circadian phase 60° (2–6 hours after the core body temperature minimum, corresponding to early biological morning). The interaction of CIRCADIAN PHASE and AGE was not significant (Fig. 4D).

There was significant interaction between FD CYCLE, TIME AWAKE, and AGE on the number of inadvertent sleeps (*F*_*8*,*11*,*310*_ = *99*.*65*, *p* < *0*.*0001*), but post hoc analysis did not show significant differences in the number of sleeps between the two age groups for any time awake bin (Fig. 5D). The interaction between FD CYCLE, CIRCADIAN PHASE, and AGE was not significant (Fig. 6D).

### Slow Eye Movements and Inadvertent Sleeps with Attentional Lapses and Subjective Sleepiness

**Figure 7:**
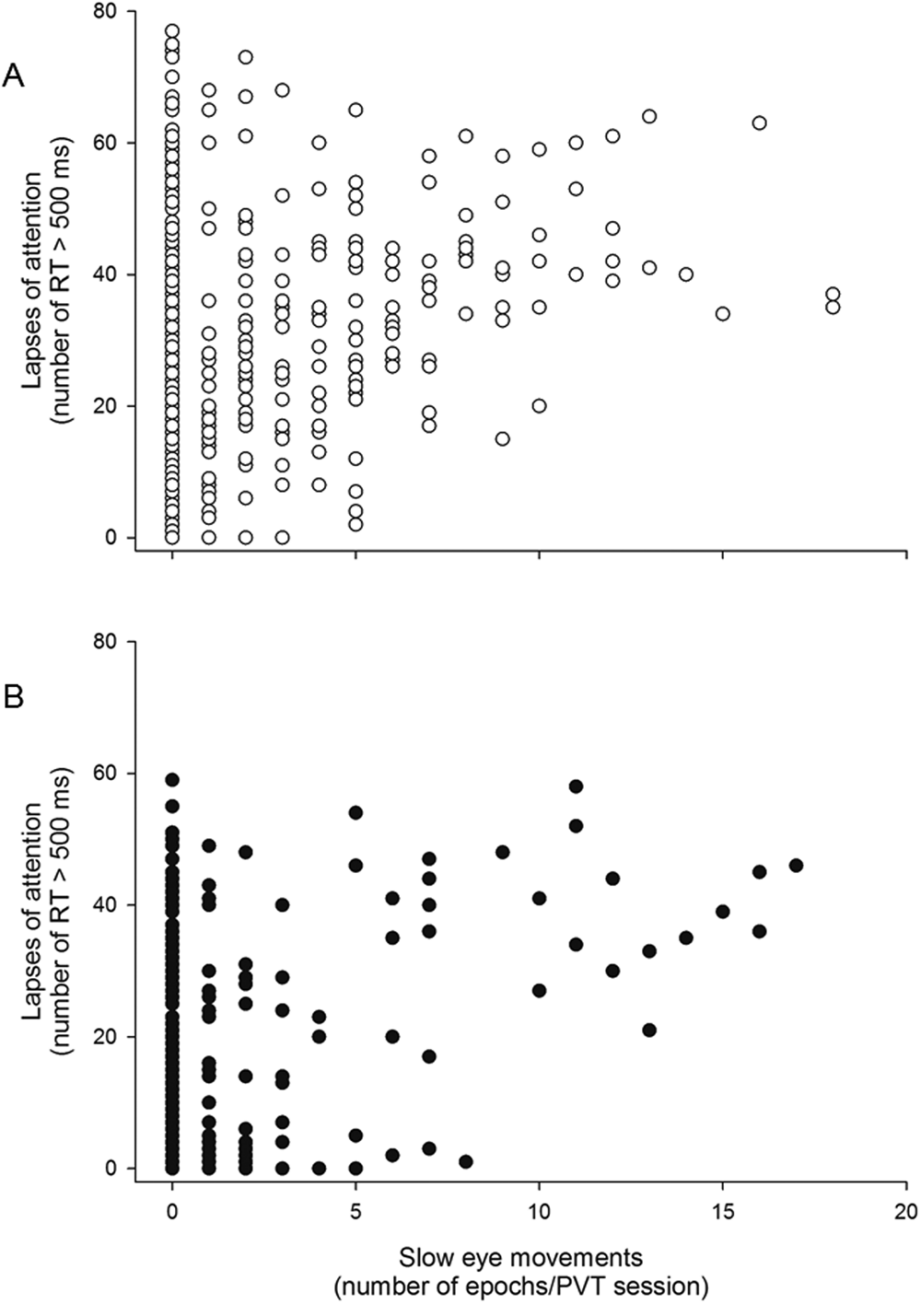
SEMs vs. lapses of attention during three weeks of FD. Each symbol represents data from one 10-minute PVT. Symbols: ○ younger participants (A; n = 12), ● older participants (B; n = 11).

There was a significant positive correlation between the number of SEMs and the number of PVT lapses during the 3 week FD among both young [*ρ* = *0*.*56*, *t*(*11*) = *6*.*32*, *p* < *0*.*0001*] and older participants [*ρ* = *0*.*27*, *t*(*10*) = *2*.*96*, *p* = *0*.*0143*; Fig. 7]. Young participants had significantly more SEMs during the 10-min PVTs (N = 12, SEM epochs per hour = 5.0 ± 0.9) than older participants (N = 11, SEM epochs per hour = 2.5 ± 1.1; p = 0.0188 two-tailed). Young participants were also significantly more likely to have at least one inadvertent sleep during their PVTs than were older participants [*χ²* (*1*, *N* = *23*) = *5*.*49*, *p* = *0*.*0191*]. Finally, there was a significant positive correlation between the number of SEMs and the KSS score during the 3 week FD among both young [*ρ* = *0*.*16*, *t*(*11*) = *5*.*65*, *p* = *0*.*0001*] and older participants [*ρ* = *0*.*17*, *t*(*11*) = *4*.*30*, *p* = *0*.*0013*], with no significant difference between the two age groups.

## Discussion

We found that while young and older adults rated themselves equally sleepy during a 3-week protocol combining sleep restriction with circadian disruption, young participants showed more neurobehavioral deficits resulting from sleepiness. Young adults had more lapses of attention and inadvertent sleep episodes, suggesting that they were significantly worse than older participants at remaining awake and sustaining attention.

Unsurprisingly, the number of inadvertent sleeps, SEMs, and PVT lapses increased with the progression of sleep restriction in both age groups, indicating reduced alertness and increased sleep pressure. There was a significant interaction between age group and duration of exposure to sleep restriction; the older participants had fewer attentional failures and inadvertent sleeps, especially during the second week of sleep restriction. A similar interaction was observed between age and time awake; while in both groups the number of lapses increased with the duration of wakefulness, older participants had fewer lapses than younger participants. Together, these findings suggest that healthy young adults are more vulnerable to chronic sleep loss than older adults, at least with respect to attention.

Our findings are consistent with reports comparing objective sleepiness and performance in response to acute sleep deprivation or chronic partial sleep loss. In earlier studies, we and others found young adults are less able than older adults to maintain their reaction time performance after 1–3 nights of sleep curtailment^20,23–25^, and after 26–40 hours of acute sleep deprivation^7,21,26^. Young adults also have more inadvertent sleep than older adults when they remain awake for 24+hours^21,22,25^.

Our observation that young adults had more inadvertent sleep and lapses of attention than older adults, yet similar number of SEMs during sleep restriction was somewhat surprising because previous studies have found parallel increases in SEMs, inadvertent sleep, PVT lapses, and PVT reaction times in young adults during extended wake episodes^27–29^. One potential explanation is that while both young and older adults become increasingly sleepy during chronic sleep restriction, sleep pressure accumulates at a faster rate in young adults. Studies in animals have found that while adenosine levels in the basal forebrain increases with age^30,31^, the sleep deprivation-induced increase in adenosine, a molecular marker of homeostatic sleep drive^32^, is either similar to or less in older animals compared to young animals after controlling for baseline levels^30,33^, suggesting a slower build-up of sleep pressure with age.

Other evidence that there is lower homeostatic sleep pressure with age includes the generally shorter sleep duration of older people. In a sleep extension study, healthy older adults slept significantly less than young adults when scheduled to stay in bed for 16 hours a day over multiple days^6^. Older adults also spend less time in SWS and exhibit a smaller absolute SWS rebound following sleep deprivation or experimental SWS suppression^2,34,35^. Furthermore, young adults show a greater sleep propensity than older adults as measured by latency to sleep in the Multiple Sleep Latency Test under rested conditions^6,34^, following total sleep deprivation^23^, and after selective SWS deprivation^34^.

An alternative explanation of our observations is that the younger and older participants have equally high sleep pressure but older adults are less able to make the transition into sleep. Age-related changes in the interaction between the homeostatic and circadian processes^36,37^, together with a decline in the number of neurons in the ventrolateral preoptic nucleus with age^38,39^, a hypothalamic switch that enables sleep-wake transitions^40^, could make it more difficult for the aging brain to transition from wakefulness to sleep. This may account for why older adults are better than young adults at maintaining their attention in reaction time tasks or in simulated driving^41^ under sleep restriction conditions. Given our finding that SEMs were more frequent among the young adults than among the older adults during the PVTs, the older adults may have been able to harness additional cognitive resources to maintain vigilance when needed^42^.

Interestingly, we found no difference in subjective sleepiness between the two age groups. Many survey studies have found increased levels of daytime sleepiness among older adults^16,17,24,43^, but often such sleepiness is associated with medical conditions, medications, or undiagnosed sleep disorders that are more frequent among older individuals^16,43,44^. In fact, studies of healthy young and older adults tend to find either no difference in subjective sleepiness, or the opposite: healthy older participants rated themselves as less sleepy than younger participants in a 2-week forced desynchrony study during which they had less sleep and poorer sleep quality^45^, during a 26-hour sleep deprivation^21^, and when time in bed was restricted to 4 hours per night for 3 days^25^.

One important finding from our study is that subjective sleepiness did not reflect objective sleepiness level in either age group. Although subjective sleepiness and SEMs both varied with time awake and circadian phase, subjective sleepiness ratings during the 3 week FD quickly leveled off and became dissociated from objective sleepiness measures, which continued to worsen. A similar dissociation during chronic sleep restriction has been observed in young adults^46,47^, suggesting that regardless of age, people are poor judges of how sleepy they are under chronic sleep loss conditions.

In healthy aging, changes also occur in the circadian system^48^. Older adults have earlier sleep times and earlier timing of their circadian rhythms^49–54^. This change towards a morning-type orientation with advancing age^50,55–57^, together with an attenuated amplitude of some circadian rhythms^58–60^ and a weakening of the circadian signal that promotes sleep in the late biological night^53,58^ with aging, may explain why older adults better maintain their performance during the early morning hours^7,45^. This finding was confirmed in our study, with older participants exhibiting fewer lapses on the PVT especially at circadian phases corresponding to the late biological night/early biological morning.

Our findings add further evidence that young adults are more vulnerable to sleep loss than healthy older adults, even when sleep deficiency occurs across three weeks of chronic sleep restriction and recurrent circadian disruption. Our finding that older adults have a greater ability to remain awake at adverse circadian phases even under chronic sleep restriction supports the hypothesis of a reduced amplitude of the circadian sleep-wake promoting signal with age. Whether there is a reduced sleep need with aging remains an open question. While the older adults in the current study were better able to remain awake and focus their attention than the young adults, they experienced the same metabolic dysfunction as the young adults as was reported previously^61^.

In summary, these results could be interpreted to suggest that young adults are biologically more vulnerable to the effects sleep loss and circadian disruption. Together with environmental and lifestyle factors (e.g., social jetlag, caffeine consumption, electronic media use) that contribute to chronic sleep loss, this greater vulnerability may help explain why the risk of sleep-related motor vehicle crashes peaks in young drivers. Thus, our results have important public health implications, particularly concerning prevention of the drowsy driving accidents in young drivers.

Limitations: We investigated the effects of three weeks of chronic sleep restriction combined with recurrent circadian disruption on subjective and objective daytime sleepiness in healthy young and older adults. By excluding people with sleep disorders and those on medication, we removed those confounding factors at the cost of generalizability. Two of the measures we used to determine objective sleepiness were based on EEG recordings, which were available for about two thirds of the scheduled wake episodes, although with minimal day-to-day variability. SEMs were scored as epochs with at least one SEM regardless of how many SEMs the epoch contained, which may have affected our ability to detect a difference between the two age groups. Finally, we could not assess whether there were episodes of local sleep that contributed to the observed age group differences.

## Methods

### Ethical approval

The Partners Health Care Human Research Committee reviewed and approved this study (2005-P-002292), which conformed to the principles outlined in the Declaration of Helsinki. Each participant gave written informed consent.

### Participants

12 young adults (mean ± SD: 22.42 ± 2.64 years; range 18–27 years; 6 female) and 12 older adults (59.58 ± 4.54 years; 55–70 years; 6 female) were recruited from the community. Participants were screened to exclude medical, psychological, and sleep disorders. Participants did not regularly take medications (with the exception of birth control) and had no acute or chronic illnesses. Screening included a medical history, physical examination, electrocardiogram, urinalysis, and clinical blood tests; psychological questionnaires (Minnesota Multiphasic Personality Inventory, Beck Depression Inventory) and a structured psychological interview with a clinical psychologist; and an all-night clinical polysomnographic (PSG) recording to rule out clinically significant sleep disorders. Participants reported no significant sleep complaints, no history of regular night shift work for at least three years, and no transmeridian travel (across more than 2 time zones) within 3 months before the study.

### Protocol

Participants maintained a regular sleep-wake schedule for at least 3 weeks before admission, with a 10-hour per night scheduled time in bed. Compliance with the sleep-wake schedule was verified by wrist actigraphy (Actiwatch-L, Mini Mitter). The 39-day studies took place in the Intensive Physiological Monitoring Unit of the Center for Clinical Investigation at Brigham and Women’s Hospital where participants were studied individually in private rooms. The laboratory environment was free of time cues, maintained at a temperature of 75 ± 3 °F, with low light levels during wake (less than 15 lux) and complete darkness during scheduled sleep opportunities. The first three days each included a 12-hour nighttime sleep opportunity and a 4-hour nap scheduled in the middle of the wake episode (Fig. 1). These sleep extension days were followed by three baseline days, each with a 10-hour night-time sleep opportunity.

Following the baseline days, the three-week CSR plus circadian disruption segment of the study took place. This consisted of 28-hour “days” consisting of a 21.47-hour wake episode and a 6.53-hour sleep opportunity (equivalent to 5.6 hours of sleep every 24 hours). This forced desynchrony (FD) protocol results in scheduled sleep episodes being distributed across the circadian cycle with similar prior wake durations^62^ and allows for the separation of the influence of sleep-wake homeostatic regulation from that of the circadian timing system, as well as the assessment of their interactions. After the three weeks of CSR-FD, there was a 10-day recovery segment with a 10-hour sleep opportunity each night (Fig. 1).

During their free time, participants were allowed to perform sedentary activities such as reading, watching movies, arts and crafts, or listening to music. During the three weeks of CSR-FD, a trained research technician was present in the participant’s room throughout each scheduled wake episode to ensure that they remained awake and adhered to the protocol.

### Data Collection and Processing

Subjective sleepiness was assessed using the KSS, which the participants took every two hours throughout their wake episodes. The KSS requires the participants to rate how sleepy they felt during the 5 minutes immediately preceding the test by selecting a number ranging from 1 (very alert) to 9 (very sleepy) that best reflects how they feel. KSS values vary with circadian phase, increase with time awake^45,63^, and are sensitive to acute sleep deprivation^21,64^.

We used a 10-min PVT to evaluate sustained attention. When taking the PVT participants were asked to respond as quickly as possible to a visual stimulus presented on a computer monitor by pressing a response button with their dominant thumb. A lapse in attention was defined by a response time over 500 milliseconds and quantified as the total number of lapses per PVT. Participants took the PVT every four waking hours. This test is sensitive to circadian phase^45,62,63^ and acute sleep loss^21,65^, while not showing any long-lasting training effects^46,66^.

PSG signals were continuously recorded throughout scheduled wake episodes with a digital recorder (Vitaport-3, TEMEC Instruments B.V., Kerkrade, The Netherlands), and included the EEG (Fz, Cz, Pz, Oz referenced to linked mastoids) and the electrooculograms (EOGs). The signals were high-pass filtered [time constants: 0.68 s (EEG, EOG)], low-pass filtered [Bessel, 24 dB/octave; −6 dB at 70.1 Hz (EEG), 34.8 Hz (EOG)], and digitized [resolution: 12 bit, sampling rate: 256 Hz (EEG), or 128 Hz (EOG)]. If the participant appeared to fall asleep during a scheduled wake episode (prolonged eye closure), research assistants verbally awakened them. To quantify physiological sleepiness, the waking EEG recordings were scored by a registered PSG technologist in 30-s epochs for the presence of SEMs (any epoch that contained at least one SEM identified as conjugate, sinusoidal deflections in the EOGs unrelated to body movements; no amplitude criterion^29^) and for the presence of inadvertent sleep (any epoch that contained sleep according to established criteria^67^). The presence of SEMs, increased EEG alpha and theta activity, and inadvertent sleep onsets during scheduled wake are measures of objective sleepiness, and their presence indicates increased sleep pressure^27–29,64,68,69^.

### Data Analysis

Data from the three 24-hour baseline days and from the eighteen 28-hour CSR-FD “days” were included in the analysis. PVT data from one older participant was omitted from analysis due to non-compliance with the testing conditions. To account for the effects of sleep inertia^70^, data from the first hour after scheduled wake time each day were excluded from analysis.

EEG/EOG recordings were available for about two-thirds (64.4 ± 2.1%, mean ± SD) of the scheduled wake episodes, with minimal day-to-day variability. The remaining third of the wake episodes included scheduled events such as showers, electrode removals, re-applications before and after the showers, and data downloads. The data were compiled into 30-minute bins, and the number of epochs with an SEM or scored as any stage of sleep was calculated for each 30-minute bin using linear extrapolation for missing epochs, and then rounded up to the nearest integer. We dichotomized the inadvertent sleep data by assigning each 30-minute bin a value 0 (no sleep epochs) or 1 (at least one sleep epoch). The 30-minute bins with less than 50% of data were excluded from analysis. For visual presentation, the SEM/SLEEP epochs in the EEG/EOG recordings were calculated and expressed as average number of epochs per hour over the selected bin.

To study the homeostatic effect of time awake on sleepiness and cognitive performance, data from each wake episode during FD was binned into five consecutive 4-hour time awake bins, beginning with scheduled wake time. This bin size was chosen to correspond to the frequency of PVT administration (once every four hours).

The intrinsic circadian period of the core body temperature data from the FD portion of the protocol was estimated for each participant using non-orthogonal spectral analysis^71^. From this estimate of intrinsic circadian period, a circadian phase (from 0 to 359°) was assigned to each minute of the study, with 0° corresponding to the minimum of the waveform fit to the entire temperature data series. To study the effect of CIRCADIAN PHASE on sleepiness and cognitive performance, data from the scheduled wake episodes were averaged into six 60-degree (~4-hour) circadian phase bins (0°, 60°, 120°, 180°, 240°, and 300°), based on each individual’s circadian period and starting phase.

To study the cumulative effect of CSR on sleepiness and cognitive performance, the data were assigned to one of three FD cycles. Each FD cycle consisted of six 28-hour “days” (equivalent to a calendar week), with each FD cycle beginning at the same clock hour and week day (Fig. 1).

To study the relationship between SEMs/inadvertent sleeps and attentional lapses during the 10-min PVTs, we calculated the number SEM/inadvertent sleep epochs within each 10-minute PVT during FD and correlated it with the total number of lapses separately for young and older participants. We also calculated the number of SEM/inadvertent sleep epochs during PVTs to better understand whether the observed age-related changes in the ability to sustain wakefulness under chronic sleep loss are uniform across wakefulness or context/task dependent.

### Statistics

Statistical analyzes were carried out using SAS version 9.4 (SAS Institute, Cary, NC). The KSS data were analyzed using Linear Mixed Models and the PVT lapse data were analyzed with Generalized Linear Models and assumed a Poisson distribution. The analyzes of SEM and inadvertent sleep data were also conducted using Generalized Linear Models, but assumed a negative binomial distribution for the SEM data and a binomial distribution for the inadvertent sleep data because these distributions best fit these data. Normality tests for residuals did not show a significant violation for any variable. Age (young vs. older), FD cycle (1, 2, 3), time awake (4, 8, 12, 16, 20 hours), and circadian phase (0°, 60°, 120°, 180°, 240°, 300°) were treated as fixed effects, with a random intercept statement incorporated into the models to allow for means to vary between participants.

We first tested whether there was a difference between the age groups at baseline by comparing data from the three baseline days between the age groups for each variable, and proceeded to drop baseline from further analysis if no significant differences between the groups were found. We then tested the main effects of age, FD cycle, time awake and circadian phase. Next, we tested all possible 2-way interactions and, where there were significant 2-way interactions among the factors FD cycle, time awake, and circadian phase, we also tested 3-way interactions by adding age as a factor. In order to avoid overfitting, higher-order interactions were not included in the models. For all significant interactions that included the factor age, we also conducted post hoc analysis by testing the simple main effect of age (between-participants factor) at each specific level of FD cycle, time awake and/or circadian phase. Thus, we were able to determine if there were differences between the young and older participants at a distinct level of the within-participants factor by testing the hypothesis that the two levels of age are equal (H0). All reported degrees of freedom and p-values are from the final statistical model for each measure. Post-hoc tests were adjusted for multiple comparisons using the Bonferroni method.

To test the strength of the relationship between the number of epochs with SEM and the number of lapses during each 10-min PVT, and between the number of epochs with SEM during the 5-minutes preceding the KSS test and the KSS score, we calculated Spearman’s rank correlation coefficient for each participant and transformed the resulting correlation coefficients (*r*-values) into z-scores using Fisher’s z-transformation. We then performed a t-test on the z-scored values to determine (within each age group) if there was a significant correlation between SEMs and lapses, or between SEMs and KSS score, by testing the hypothesis that the population correlation ρ is zero (H_0_: ρ = 0; H_1_: ρ ≠ 0). Inverse z-transformation was used on the average of the z-scored values to quantify the overall effect size (ρ). Finally, we performed Wilcoxon rank-sum test to test the equality of the two population correlations (young vs. older) by testing the hypothesis that the two population correlations are equal (H_0_: ρ_1_ = ρ_2_; H_1_: ρ_1_ ≠ ρ_2_). Because most of the older participants had no inadvertent sleeps during their PVTs, we did not conduct a correlation analysis between the number of inadvertent sleeps and the number of PVT lapses. Instead, we performed a Chi-Square test to examine whether having at least one inadvertent sleep during the PVTs was more frequent among the young than the older participants.

All results are reported as mean ± SD unless otherwise indicated. The critical significance level was set to α = 0.05 for all tests. Note that because significant interactions in the statistical models vary between measures, degrees of freedom are not uniform.

### Data availability

The authors will make de-identified data from the current study available upon written request. Execution of a Materials Transfer Agreement is required if the data will be used in research supported by a for-profit company, per Partners Healthcare Institutional Review Board policy. The specific data to be shared will be: KSS scores, number of PVT lapses (RTs > 500 milliseconds) per session, number of SEM epochs and inadvertent SLEEP epochs per 30 minutes, with the associated timing information, from the 3 baseline and 21 FD wake episodes as described in the methods section.

## References

[1] Bliwise, D. L. In Principles and Practice of Sleep Medicine Vol. Fourth (eds M. H. Kryger, T. Roth, & W. C. Dement) 24–38 (Elsevier Saunders, 2005).

[2] Dijk DJ, Duffy JF, Czeisler CA (2000). Contribution of circadian physiology and sleep homeostasis to age-related changes in human sleep. Chronobiol Int.

[3] Ohayon MM, Carskadon MA, Guilleminault C, Vitiello MV (2004). Meta-analysis of quantitative sleep parameters from childhood to old age in healthy individuals: developing normative sleep values across the human lifespan. Sleep.

[4] Miner B, Kryger MH (2017). Sleep in the Aging Population. Sleep Medicine Clinics.

[5] Borbély AA (1982). A two process model of sleep regulation. Human Neurobiology.

[6] Klerman EB, Dijk DJ (2008). Age-related reduction in the maximal capacity for sleep–implications for insomnia. Current biology: CB.

[7] Adam M, Retey JV, Khatami R, Landolt HP (2006). Age-related changes in the time course of vigilant attention during 40 hours without sleep in men. Sleep.

[8] Dijk DJ, Kelly TK, Riel E, Duffy JF, Czeisler CA (1999). Altered homeostatic delta EEG response to sleep loss in older people?. Sleep.

[9] Carskadon MA, Dement WC (1987). Daytime sleepiness: Quantification of a behavioral state. Neuroscience and Biobehavioral Reviews.

[10] Cajochen C, Munch M, Knoblauch V, Blatter K, Wirz-Justice A (2006). Age-related changes in the circadian and homeostatic regulation of human sleep. Chronobiology International.

[11] Mander BA, Winer JR, Walker MP (2017). Sleep and Human Aging. Neuron.

[12] Skeldon AC, Derks G, Dijk DJ (2016). Modelling changes in sleep timing and duration across the lifespan: Changes in circadian rhythmicity or sleep homeostasis?. Sleep Med Rev.

[13] Bonnet MH (1986). Performance and sleepiness following moderate sleep disruption and slow wave sleep deprivation. Physiology and Behavior.

[14] Gillberg M, Åkerstedt T (1994). Sleep restriction and SWS-suppression: effects on daytime alertness and night-time recovery. J Sleep Res.

[15] Walsh JK, Hartman PG, Schweitzer PK (1994). Slow-wave sleep deprivation and waking function. J Sleep Res.

[16] Prinz PN, Vitiello MV, Raskind MA, Thorpy MJ (1990). Geriatrics: Sleep disorders and aging. N. Engl. J. Med.

[17] Feinsilver, S. H. Sleep in the elderly. What is normal? *Clin Geriatr Med* **19**, 177–188, viii (2003). 10.1016/s0749-0690(02)00064-2 12735121

[18] Bonnet MH (1989). The effect of sleep fragmentation on sleep and performance in younger and older subjects. Neurobiology of Aging.

[19] O’Donnell D (2009). Comparison of subjective and objective assessments of sleep in healthy older subjects without sleep complaints. J Sleep Res.

[20] Bonnet MH, Rosa RR (1987). Sleep and performance in young adults and older normals and insomniacs during acute sleep loss and recovery. Biol Psychol.

[21] Duffy JF, Willson HJ, Wang W, Czeisler CA (2009). Healthy older adults better tolerate sleep deprivation than young adults. Journal of the American Geriatric Society.

[22] Buysse DJ (1993). Patterns of sleep episodes in young and elderly adults during a 36-hour constant routine. Sleep.

[23] Brendel DH (1990). Sleep stage physiology, mood, and vigilance responses to total sleep deprivation in healthy 80-year-olds and 20-years-olds. Psychophysiology.

[24] Philip P (2004). Age, performance and sleep deprivation. J Sleep Res.

[25] Stenuit P, Kerkhofs M (2005). Age modulates the effects of sleep restriction in women. Sleep.

[26] Smulders FT, Kenemans JL, Jonkman LM, Kok A (1997). The effects of sleep loss on task performance and the electroencephalogram in young and elderly subjects. Biol Psychol.

[27] Cajochen C, Khalsa SBS, Wyatt JK, Czeisler CA, Dijk DJ (1999). EEG and ocular correlates of circadian melatonin phase and human performance decrements during sleep loss. American Journal of Physiology.

[28] Wyatt JK, Cajochen C, Ritz-De Cecco A, Czeisler CA, Dijk DJ (2004). Low-dose repeated caffeine administration for circadian-phase-dependent performance degradation during extended wakefulness. Sleep.

[29] Grady S, Aeschbach D, Wright KP, Czeisler CA (2010). Effect of modafinil on impairments in neurobehavioral performance and learning associated with extended wakefulness and circadian misalignment. Neuropsychopharmacology.

[30] Murillo-Rodriguez E, Blanco-Centurion C, Gerashchenko D, Salin-Pascual RJ, Shiromani PJ (2004). The diurnal rhythm of adenosine levels in the basal forebrain of young and old rats. Neuroscience.

[31] Mackiewicz M (2006). Age-related changes in adenosine metabolic enzymes in sleep/wake regulatory areas of the brain. Neurobiology of Aging.

[32] Porkka-Heiskanen T (1997). Adenosine: A mediator of the sleep-inducing effects of prolonged wakefulness. Science.

[33] Rytkonen KM, Wigren HK, Kostin A, Porkka-Heiskanen T, Kalinchuk AV (2010). Nitric oxide mediated recovery sleep is attenuated with aging. Neurobiology of Aging.

[34] Dijk DJ, Groeger JA, Stanley N, Deacon S (2010). Age-related reduction in daytime sleep propensity and noctural slow wave sleep. Sleep.

[35] Munch M (2004). The frontal predominance in human EEG delta activity after sleep loss decreases with age. Eur.J Neurosci.

[36] Munch M, Knoblauch V, Blatter K, Wirz-Justice A, Cajochen C (2007). Is Homeostatic sleep regulation under low sleep pressure modified by age?. Sleep.

[37] Dijk DJ, Duffy JF, Riel E, Shanahan TL, Czeisler CA (1999). Ageing and the circadian and homeostatic regulation of human sleep during forced desynchrony of rest, melatonin and temperature rhythms. J Physiol (Lond).

[38] Hofman MA, Swaab DF (1989). The sexually dimorphic nucleus of the preoptic area in the human brain: a comparative morphometric study. J Anat..

[39] Lim AS (2014). Sleep is related to neuron numbers in the ventrolateral preoptic/intermediate nucleus in older adults with and without Alzheimer’s disease. Brain: a journal of neurology.

[40] Saper CB, Fuller PM, Pedersen NP, Lu J, Scammell TE (2010). Sleep state switching. Neuron.

[41] Filtness, A. J., Reyner, L. A. & Horne, J. A. Driver sleepiness-comparisons between young and older men during a monotonous afternoon simulated drive. *Biol Psychol* **89** (2012). 10.1016/j.biopsycho.2012.01.002 22266164

[42] Grady C (2012). The cognitive neuroscience of ageing. Nature reviews. Neuroscience.

[43] Whitney CW (1998). Correlates of daytime sleepiness in 4578 elderly persons: the Cardiovascular Health Study. Sleep.

[44] Foley D, Ancoli-Israel S, Britz P, Walsh J (2004). Sleep disturbances and chronic disease in older adults: results of the 2003 National Sleep Foundation Sleep in America Survey. J Psychosom.Res.

[45] Silva EJ, Wang W, Ronda JM, Wyatt JK, Duffy JF (2010). Circadian and wake-dependent influences on subjective sleepiness, cognitive throughput, and reaction time performance in older and young adults. Sleep.

[46] Van Dongen HPA, Maislin G, Mullington JM, Dinges DF (2003). The cumulative cost of additional wakefulness: Dose-response effects on neurobehavioral functions and sleep physiology from chronic sleep restriction and total sleep deprivation. Sleep.

[47] Lee JH (2009). Neurobehavioral performance in young adults living on a 28-h day for 6 weeks. Sleep.

[48] Duffy JF, Zitting KM, Chinoy ED (2015). Aging and Circadian Rhythms. Sleep Medicine Clinics.

[49] Carrier J, Monk TH, Reynolds CF, Buysse DJ, Kupfer DJ (1999). Are age differences in sleep due to phase differences in the output of the circadian timing system?. Chronobiol Int.

[50] Czeisler CA (1992). Association of sleep-wake habits in older people with changes in output of circadian pacemaker. Lancet.

[51] Monk TH, Buysse DJ, Reynolds CF, Kupfer DJ, Houck PR (1995). Circadian temperature rhythms of older people. Exp Gerontol.

[52] Prinz PN (1984). Circadian temperature variation in healthy aged and in Alzheimer’s disease. Journal of Gerontology.

[53] Duffy JF, Dijk DJ, Klerman EB, Czeisler CA (1998). Later endogenous circadian temperature nadir relative to an earlier wake time in older people. American Journal of Physiology.

[54] Duffy JF, Czeisler CA (2002). Age-related change in the relationship between circadian period, circadian phase, and diurnal preference in humans. Neurosci Lett.

[55] Adan A (2012). Circadian typology: A comprehensive review. Chronobiology International.

[56] Monk TH (1991). Circadian characteristics of healthy 80-year-olds and their relationship to objectively recorded sleep. J Gerontol.

[57] Merikanto I (2012). Relation of chronotype to sleep complaints in the general Finnish population. Chronobiology International.

[58] Dijk DJ, Duffy JF (1999). Circadian regulation of human sleep and age-related changes in its timing, consolidation and EEG characteristics. Ann Med.

[59] Weitzman ED, Moline ML, Czeisler CA, Zimmerman JC (1982). Chronobiology of aging: Temperature, sleep-wake rhythms and entrainment. Neurobiol Aging.

[60] Carrier J, Monk TH, Buysse DJ, Kupfer D (1996). Amplitude reduction of the circadian temperature and sleep rhythms in the elderly. Chronobiol Int.

[61] Buxton OM (2012). Adverse metabolic consequences in humans of prolonged sleep restriction combined with circadian disruption. Science Translational Medicine.

[62] Dijk DJ, Czeisler CA (1994). Paradoxical timing of the circadian rhythm of sleep propensity serves to consolidate sleep and wakefulness in humans. Neurosci Lett.

[63] Wyatt JK, Ritz-De Cecco A, Czeisler CA, Dijk DJ (1999). Circadian temperature and melatonin rhythms, sleep, and neurobehavioral function in humans living on a 20-h day. Am J Physiol Regul Integr Comp Physiol.

[64] Åkerstedt T, Gillberg M (1990). Subjective and objective sleepiness in the active individual. Int J Neurosci.

[65] Dorrian, J., Rogers, N. L. & Dinges, D. F. In *Sleep Deprivation*. *Clinical Issues*, *Pharmacology*, and Sleep Loss Effects (ed. Kushida, C. A.) 39–70 (Marcel Dekker, 2005).

[66] Dinges DF, Powell JW (1985). Microcomputer analyses of performance on a portable, simple visual RT task during sustained operations. Behavior Research Methods, Instruments & Computers.

[67] Rechtschaffen, A. & Kales, A. *A manual of standardized terminology*, *techniques and scoring system for sleep stages of human Subjects*. (U.S. Government Printing Office, 1968). 10.1046/j.1440-1819.2001.00810.x 11422885

[68] Torsvall L, Åkerstedt T (1988). Extreme sleepiness: Quantification of EOG and spectral EEG parameters. Int J Neurosci.

[69] Torsvall L, Åkerstedt T (1987). Sleepiness on the job: Continuously measured EEG changes in train drivers. Electroencephalogr.Clin Neurophysiol..

[70] Silva EJ, Duffy JF (2008). Sleep inertia varies with circadian phase and sleep stage in older adults. Behav Neurosci.

[71] Czeisler CA (1999). Stability, precision, and near-24-hour period of the human circadian pacemaker. Science.

